# Raised Cecal Veillonella (Firmicutes)/S 24-7 (Bacteriodetes) May Not Cause Salt-Sensitive Hypertension

**DOI:** 10.3389/fphys.2016.00118

**Published:** 2016-03-31

**Authors:** Arun Chaudhury

**Affiliations:** GIM FoundationLittle Rock, AR, USA

**Keywords:** microbiome, hypertension, causal link

“They” were always there. Or at least, “they” were there since antiquity; since the time that foraging for food in the wild invariably meant consuming “them” laced on the food. In the hollow organs and on the surface, “they” live peacefully, in harmony. “They”: The “Microbiome.” Trillions of “them”! (Sonnenburg et al., 2004; Bäckhed et al., 2005; Pluznick, 2014; Lanza et al., 2015; McNally and Brown, 2015; Mermel, 2015; Woolhouse et al., 2015) When the balance gets tipped off, at least in some organs, clinical signs are manifested. For example, *Gardnerella*, a common constituent of vaginal micro-organisms, colonize and smear the epitheliocyte producing the so-called “clue cells,” and manifest a local secretory phenome, along with odorous volatile substances production, the “whiff,” so commonly detected during presentations of bacterial vaginosis (BV) in the STI clinic (Machado and Cerca, 2015). “They” love the lack of oxygen.

The greatest fermentor in the human body is the post-esophageal gut; the anaerobic environment is capable of producing an environment where gases may be formed, both in the foregut, through the small intestines, and in the hindgut, with the gradient of bioreactor activity increasing in that order. We are all so accustomed with the “wind.” Though the precise biological significance of these luminal gas productions is not known (Azpiroz, 2005), in the surgical ward, especially after abdominal laparotomy, the history of the passed “wind” is a tale of relief, a sure sign that the dreaded risk for the development of ileus has passed (Cosyns et al., 2015).

In the foregut, especially in the stomach, casual clinical correlation has been demonstrated between the presence of *Helicobacter pylori* and development of gastric ulcers (Marshall and Warren, 1984). Though the organism has been isolated form the stomach, direct evidence that their colonization is the first event in development of gastric ulcers has never been provided (Beasley et al., 2015). Furthermore, recent evidence show that *Helicobacter* has co-evolved in the human stomach for several million years and may actually play symbiotic roles (Blaser, 2012; Sitaraman, 2015). In fact, urease producing organisms have been recently demonstrated even in the small intestines, where the pH is relatively higher (near the alkaline range), and thus there may be special chemical roles, yet undefined, of urease producing organisms in the alkaline intestinal luminal environment (Shen et al., 2015).

My reason for this prelude is to generate discussion regarding some recent reports that have attempted to propose causative link between microbiome populations and their cohort changes as mechanistic basis for hypertension, continuing the recent trend of linking microbiome organizations or alterations to chronic diseases. One of this is a recent well-performed study that endeavors to establish a relation between altered cecal populations of microbes in Dahl salt sensitive rats as the basis for their hypertension (Mell et al., 2015). I present an alternate viewpoint that the microbiome changes reported in this manuscript may not necessarily be causative. Previous studies have proposed the role of gut microbiota and metabolite sensing by G coupled receptors in kidney (Pluznick et al., 2013). Oral administration of *Lactobacillus*-fermented milk reduce blood pressure in spontaneously-hypertensive rats (SHR) and humans, and it has been suggested that this may be mediated by short peptide sequences from bacterial metabolites (Yamamoto et al., 1994; Nakamura et al., 1995; Seppo et al., 2003; Sekirov et al., 2010; Khalesi et al., 2014). Other studies have shown that the gut microbes metabolize phospholipid to TMAO, and this may predispose to cardiovascular disease (Wang et al., 2011). However, TMAO can be well-metabolized (Barrett and Kwan, 1985) and how this suggested metabolite predispose to cardiovascular stress is not well established.

In this recent report (Mell et al., 2015), 16S rRNA gene sequencing obtained from fecal samples of Dahl salt-sensitive (S) and Dahl salt resistant (R) rats demonstrated that the phylum Bacteriodetes (family S24-7) and Firmicutes (family Vellionellaceae) were higher in the S rats compared with the R rats. Both strains were subjected to the following protocols: (i) maintained on a high-salt diet (ii) antibiotics treatment for ablation of microbiota and (iii) transplanted with S or R rat cecal contents. Blood pressure (BP) monitoring was performed under the pre-described circumstances.

Surprisingly, antibiotic ablation of gut microbiota of S rats did not cause significant decrease of blood pressure in comparison to R rats (Mell et al., 2015). This may support the inference that colonic microbiota and/or its metabolites may not play fundamental role in blood pressure elevation in S rats. Alternatively, we also do not know whether the microbiota resulted in overgrowth of another intraluminal non-targeted species. The systolic BP of the R rats remained unchanged when S rat cecal content was transplanted by trans-gastric gavage. Reciprocally, when S rats were administered a single bolus of cecal content from R rats, the systolic BP did not decrease but showed sustained hypertension during their entire lifespan (Mell et al., 2015). The temporal profile of the development of high BP after transplantation of the R contents to S rats was almost similar to the native (untreated) S strain. These observations indicate that this may have resulted from the existing pathology in S rats rather than induced by microbiota transplant. R rats resist development of high blood pressure even when fed a high salt (8% NaCl) diet. The authors suggest that the R cecal transplant induced accelerated hypertension (Mell et al., 2015). If such is truly the case, then the toxic components of the R ceca merits to be identified in future studies. Contrary to the thesis that the authors outline in their report (Mell et al., 2015), these key observations suggest that (i) the cecal microbiota and metabolites of S strain do not contribute to the development of hypertension and (ii) the R strain cecal microbiota/metabolites potentially restrict development of hypertension. Rather, the polygenic deregulation of smooth muscles functions contributing to arteriolar resistance and other humoral factors that regulate electrolyte homeostasis and maintenance of normal blood pressure are the potential contributing factors to development of hypertension in the salt-sensitive S strain of rats and resistance to development of hypertension in the salt-resistant R stain of rats (Rapp, 2000; Zicha et al., 2012).

We shall look forward to validatory studies for the preliminary observations made in the report. After transplantation of R contents in S rats, the demise of the rats occurred earlier than regular S rats (Mell et al., 2015). Necropsy findings, especially of gross heart morphology, may provide important data regarding the cause of accelerated mortality (for example, if development of heart failure due to aggravated hypertension). The R_cecal_ to S transplanted group demonstrated increased plasma acetate and heptanoate but lower levels of fecal Vellionellaceae (unlike the native S rats; Mell et al., 2015). *Veillonella* ferments lactate to acetate via methylmalonyl-CoA pathway:

$$\text{Lactate}\to \text{acetate}+2\text{propionate}+{\text{CO}}_{2}+{\text{H}}_{2}\text{O}$$

How acetate remained elevated despite decrease of *Veillonella* and the contribution of other organisms, especially the short-chain fatty acid (butyrate) producing biomass (Duncan et al., 2004), merits systemic examination in future studies.

Metabonomic studies have provided understanding of gut metabolism of drugs like simvastatin, one the most commonly prescribed statins (Aura et al., 2011). Simvastatin is an inhibitor of the rate-limiting cholesterol synthetic enzyme, 3-hydroxy-3-methylglutaryl coenzyme A. Simvastatin is degraded in the gut by hydrolytic cleavage of methylbutanoic acid from the backbone structure. Metabolism involves sequential microbial processes of demethylation of dimethylbutanoic acid and subsequent hydroxylation/dehydroxylation and β-oxidation, resulting in the production of short chain organic acids like 2-hydroxyisovaleric acid (3-methyl-2-hydroxybutanoic acid), 3-hydroxybutanoic acid and lactic acid (2-hydroxypropanoic acid), and finally re-cyclization of heptanoic acid to produce cyclohexanecarboxylic acid. These metabolites may have positive impact on vascular health. The increased circulating heptanoic acid in S animals transplanted with R cecal contents (Mell et al., 2015) may actually be a beneficial metabolite which may contribute to improved vascular function, rather than contributing to hypertension. This important aspect merits clarification in future studies. These aspects are also of importance in HIV infected patients who are on chronic ARTs. Some classes of HAART agents like protease inhibitors and non-nucleoside reverse transcriptase inhibitors (NNRTIs) predispose to hypercholesterolemia, hypertension and other dysmetabolic features (da Cunha et al., 2015). Administration of statins merits careful selection, for example, pravastatin over other class representatives (in order to prevent statin-induced myositis and other complications like rhabdomyolysis; Martínez et al., 2004). How the gut microbes influences such luminal drug metabolism are important areas of systemic investigations.

The metabolic role of the luminal microorganisms are complex, and how they contribute to salt regulation should be an important area of ongoing studies. Absorption of sodium chloride from the intestine is complex, and occurs as a biphasic phenomenon: (a) from the small intestine and (b) during the entire colonic transit, including in the cecum. This is further dependent on ileocecal delivery rates, colonic transit time and state of luminal electrolyte composition and viscous state of the luminal mass. In this aspect, studying the mucin metabolizing organism *Akkermansia muciniphila* may also hold important implications (Dao et al., 2015). In the animal kingdom, not all species have the cecum, for example, the sloth and the hippopotamus. The sloth is remarkable that it is a foregut fermentor (Gilmore et al., 2001), like some bird species like hoatzins and some other animals with complex stomach compartments belonging to the family Artiodactyla (suborder Ruminantia, about 150 species; Munn et al., 2008; Godoy-Vitorino et al., 2012). Other animals that have mechanism of foregut fermentation include macropods (kangaroos, tree-kangaroos, wallabies and pademelons) and potoroids (bettongs, potoroos and rat-kangaroos; Smith, 2009), which demonstrate a form of rumination called merycism. Folivorous black-tailed tree rat (*Thallomys migricauda*) surviving on acacia tree also shows foregut microorganisms. The foregut microbiota may play complex roles, including thermoregulation in mousebirds, providing luminal lysozyme to kill pathogenic bacteria acquired during foraging or simply toxin regulation, for example contributing to oxalate metabolism. These organisms also play major role in nutrient extraction (Ali Shah et al., 2014). It has also been examined that there is an inverse relationship between the volume of stomach, especially in foregut fermentors, and volume of cecum in the hindgut (Langer and Snipes, 1991). These animal species may provide important control conditions to evaluate the role of cecal organisms and their changes in hypertension, and whether foregut fermentation may provide beneficial effects for blood pressure regulation. A provocative hypothesis could be that the foregut organisms may play major role in carbon balance and the production of gases (CO2 + CH4, carbon dioxide and methane, respectively) may be nature's way to prevent absorption of excess carbon units and thus maintain body mass balance. The critical role of methanogenic bacteria in such context acquires importance.

There may be complex relationships contributing to the transit of colonic contents, salt absorption, volume expansion and contribution to regulation of BP. In my opinion, the most significant observation in the present study (Mell et al., 2015) is the change of microbiota composition of the untreated S rats: the increase in Firmicutes (“tough skin”) and Bacteriodetes. A previous study reported changes in bacterial super kingdom level in a mouse obesity model (Ley et al., 2005). Because obesity potentially may elevate blood pressure, these reverse changes (increased Firmicutes in comparison to Bacteroidetes in obesity gut microbiome) from the observations of (Mell et al., 2015) should be reconciled in future studies. There are other recent studies that suggest a role of gut microbiome in sleep apnea induced hypertension (Durgan et al., 2016); however, diet is an important variable that do not support the role of the microbiome exclusively to produce the phenotypic effects (OSA-related hypertension). Detailed metabolic examination of these two bacterial families may help to understand the gut luminal environmental contribution to salt balance. The study by Mell et al. (2015) is a timely stimulus for active investigation in the field: the role of different diets (carnivorous, herbivorous, omnivorous and low-salt) and effects on blood pressure regulation. The findings of the present study may find future merit in biotechnological innovations. For example, luminal halophiles that cause morbid infections like Vibrios may be managed with a probiotic designed on the cecal composition of R strain of rats (presuming these organisms have the ability to scavenge sodium). We need more studies that progress beyond mere cataloging of the microbiome changes to important chronic medical conditions like obesity, hypertension and post-infectious irritable bowel syndrome (IBS). An important control to incorporate during these studies is the responses of the proposed microbiome using gnotobiotic and germ-free animals (Shroff and Cebra, 1995; Cebra et al., 1998). These future investigations shall provide insights regarding the homeostatic role of the microbiome in health and mechanistic contribution(s) of their alterations in disease pathophysiology.

The microorganisms have co-stayed with human host for millions of years. During disease processes, the Nash equilibrium between the microbiome and the human host may be reset, possibly resulting in alterations of specific forms (Cho and Blaser, 2012). The most pressing issue remains how to ask the relevant reductionist questions, and how these questions may be turned to relevant pharmacobiotic products. Similar repertoire of microbiome changes have been demonstrated in other models of hypertension. For example, increase in fecal Firmicutes/Bacteriodetes ratios have been recently demonstrated in spontaneously hypertensive rats and pharmacologically induced hypertension (Yang et al., 2015). Interestingly, these same changes were also demonstrated in stool samples of a very small series of human hypertensive subjects, and the trend reversed by anti-hypertensive drugs (Yang et al., 2015). Gut metabolism of catecholamines may have a biological impact on blood pressure (Honour, 2015) and during use of antihypertensives. These aspects should guide future studies. Deep sequencing of human stool samples of known-cause and essential human hypertension subjects is a logical and significant next set of studies. Correlation of clinical conditions like small intestinal bacterial overgrowth (SIBO) in irritable bowel syndrome (IBS) and liver diseases, cystic fibrosis, diabetes mellitus-associated hypertension, ESRD patients, nosocomial infections, milk intolerance, early-life antibiotic exposure, antibiotic-associated diarrhea and aging, microbiome/metabolite changes and possible effects on blood pressure regulation may be clinically investigated. The translational avenue that merits logical expansion of these pilot observations are the benefits from all these research that may be brought upfront to the clinic. Systemic clinical studies may test the role of probiotics like *Saccharomyces boulardii* or *Lactobacillus* or a fiber-rich diet in alleviating hypertension. Several cultures around the world favor fermented milk or organic acid-based drinks, from the inner plains of Mongolia through rest of the world: in the form of kefir, dahi, various forms of cheese, yogurt, Evolus, Calpis milk or kombucha. Basic studies shall help distinguish whether the reported microbiome changes in hypertension (Mell et al., 2015; Yang et al., 2015) are simply a bystander effect and response of microbiome to microenvironmental changes, or a deeper role as a precedent event to the development of hypertension. Preliminary studies have suggested that the fetus-in-delivery acquires maternal *Lactobacillus johnsonii* in preparation for digestion of maternal milk (Aagaard et al., 2012). Future shall tell us whether old therapies like fecal enemas (Eiseman et al., 1958) shall come of age as significant adjunct pharmacotherapy(s) for management of hypertension, the major cause of all-cause morbidity with advancing age. These studies (Mell et al., 2015; Yang et al., 2015) are providing incipient evidence that Paleolithic habits, such as those followed by Hadza tribes (Schnorr et al., 2014), may enhance longevity by delaying cardiovascular morbidity. Simple sharing of Metachnikoff's advice of the beneficial effects of milk and yogurt (Metchnikoff, 1910) shall be additive to the pharmacomanagement of subjects seeking continued care for essential hypertension and may provide basis for reducing the challenges of polypharmacy.

## Author contributions

The author confirms being the sole contributor of this work and approved it for publication.

### Conflict of interest statement

The author declares that the research was conducted in the absence of any commercial or financial relationships that could be construed as a potential conflict of interest.
